# Drug Repurposing for Targeting Acute Leukemia With *KMT2A* (*MLL*)—Gene Rearrangements

**DOI:** 10.3389/fphar.2021.741413

**Published:** 2021-09-14

**Authors:** Alexia Tsakaneli, Owen Williams

**Affiliations:** Cancer Section, Developmental Biology and Cancer Programme, Great Ormond Street Institute of Child Health, University College London, London, United Kingdom

**Keywords:** MLL-rearrangements, leukemia, drug repurposing, AML, ALL

## Abstract

The treatment failure rates of acute leukemia with rearrangements of the Mixed Lineage Leukemia (MLL) gene highlight the need for novel therapeutic approaches. Taking into consideration the limitations of the current therapies and the advantages of novel strategies for drug discovery, drug repurposing offers valuable opportunities to identify treatments and develop therapeutic approaches quickly and effectively for acute leukemia with *MLL*-rearrangements. These approaches are complimentary to de novo drug discovery and have taken advantage of increased knowledge of the mechanistic basis of MLL-fusion protein complex function as well as refined drug repurposing screens. Despite the vast number of different leukemia associated *MLL*-rearrangements, the existence of common core oncogenic pathways holds the promise that many such therapies will be broadly applicable to *MLL*-rearranged leukemia as a whole.

## Introduction

Drug repurposing or drug repositioning refers to the use of an already clinically approved or experimental drug to a condition different from the one that the drug was originally developed for (Ashburn and Thor, 2004). It is not surprising that more than one molecular target can potentially be affected by most small molecules. Drug repurposing tackles two main problems of the research development process in the pharmaceutical industry: cost and time. *De novo* drug discovery and development of a drug for cancer takes approximately 10–17 years, with almost half of this time elapsing before the start of phase I clinical trials (Zhang et al., 2020). As important as time, the cost of bringing new drugs into the market is estimated to be between 2 and 3 billion USD on average. Even though the cost has increased with time, and scientific and technological factors ought to make the process of drug development faster and more efficient, the number of drugs approved every year per USD spent on development has halved roughly every 9 years since the 1950s (Munos, 2009; Scannell et al., 2012; Nosengo, 2016). In addition to this, the possibly most important advantage of drug repurposing is the lower risk of failure due to adverse effects. The safety of a drug that is being repurposed is already known through its clinical application, therefore it is much less likely for it to fail due to unexpected toxicity. The methodologies used to repurpose different therapeutic agents can be hypothesis driven or explorative and they include both computational and experimental approaches. *In silico* approaches are used to analyze different large-scale datasets, such as genome wide association studies (GWAS), signature matching, computational molecular docking and pathway or network mapping (Pushpakom et al., 2019). In parallel, phenotypic screening and binding assays such as affinity chromatography and mass spectrometry are used to experimentally identify the targets and off-targets of drugs in the lab (Martinez Molina et al., 2013; Moffat et al., 2017). Drug repurposing has already had several success stories. One of the most impressive examples in the field is the repurposing of thalidomide, a sedative that was given to pregnant women suffering from morning sickness with tragic skeletal birth defects. Thalidomide is now used for erythema nodosum leprosum and has achieved excellent results against multiple myeloma (Ashburn and Thor, 2004; Palumbo et al., 2008). In preventive care, the repurposing of aspirin, originally administered as an analgetic, is now helping prevent cardiovascular disease and colorectal cancer (Ishida et al., 2016). More recently, the SARS-CoV-2 emergency created the need for treatments for patients suffering from COVID-19. To this end, many labs screened for agents with the potential to be repurposed for COVID-19 patients and identified among others broad-spectrum antiviral agents (BSAAs) originally approved for Influenza or HIV and immunomodulatory and anti-inflammatory agents, such as the human IL-6 receptor (IL-6R) inhibitor tocilizumab as potential treatments for COVID-19 (Scavone et al., 2020; Singh et al., 2020).

In acute leukemia, 11q23 chromosomal rearrangements, affecting the *MLL* (*KMT2A*) gene, are a genetic hallmark and one of the major driver mutations (Meyer et al., 2018). This class of abnormalities is associated with 70–80% of infant acute leukemias, 15–20% of pediatric AML and is also found less frequently in adult patients (Hilden et al., 2006; Pieters et al., 2007; Harrison et al., 2010). The prognosis for these patients is poor. For example, *MLL*-rearranged infant ALL 5-years event-free survival (EFS) is only 20–50%, in comparison to over 60% for those with wild-type MLL (Hilden et al., 2006; Pieters et al., 2019). For young adult ALL patients, despite very high initial remission rates of 80–90%, unfortunately 50% of these patients will eventually relapse. Similarly, in older patients, initial remission is achieved in 40–90% of the patients with only 10–20% of them reaching a 5-years overall survival rate (Guru Murthy et al., 2015; Winters and Bernt, 2017). *MLL*-rearranged leukemias are not treated under a distinct protocol, but mainly on regimens based on the disease lineage (AML or ALL) and the age of the patient. The standard treatment typically consists of induction chemotherapy, followed by intensive chemotherapy followed by additional therapy. Over 130 different *MLL* translocations with numerous different fusion partners have been described, although only a fraction of these are recurrent (Meyer et al., 2018). Development of novel therapies for *MLL*-rearranged leukemia should take this oncogenic diversity into account and ensure broad applicability by targeting core elements of this disease.

**FIGURE 1:**
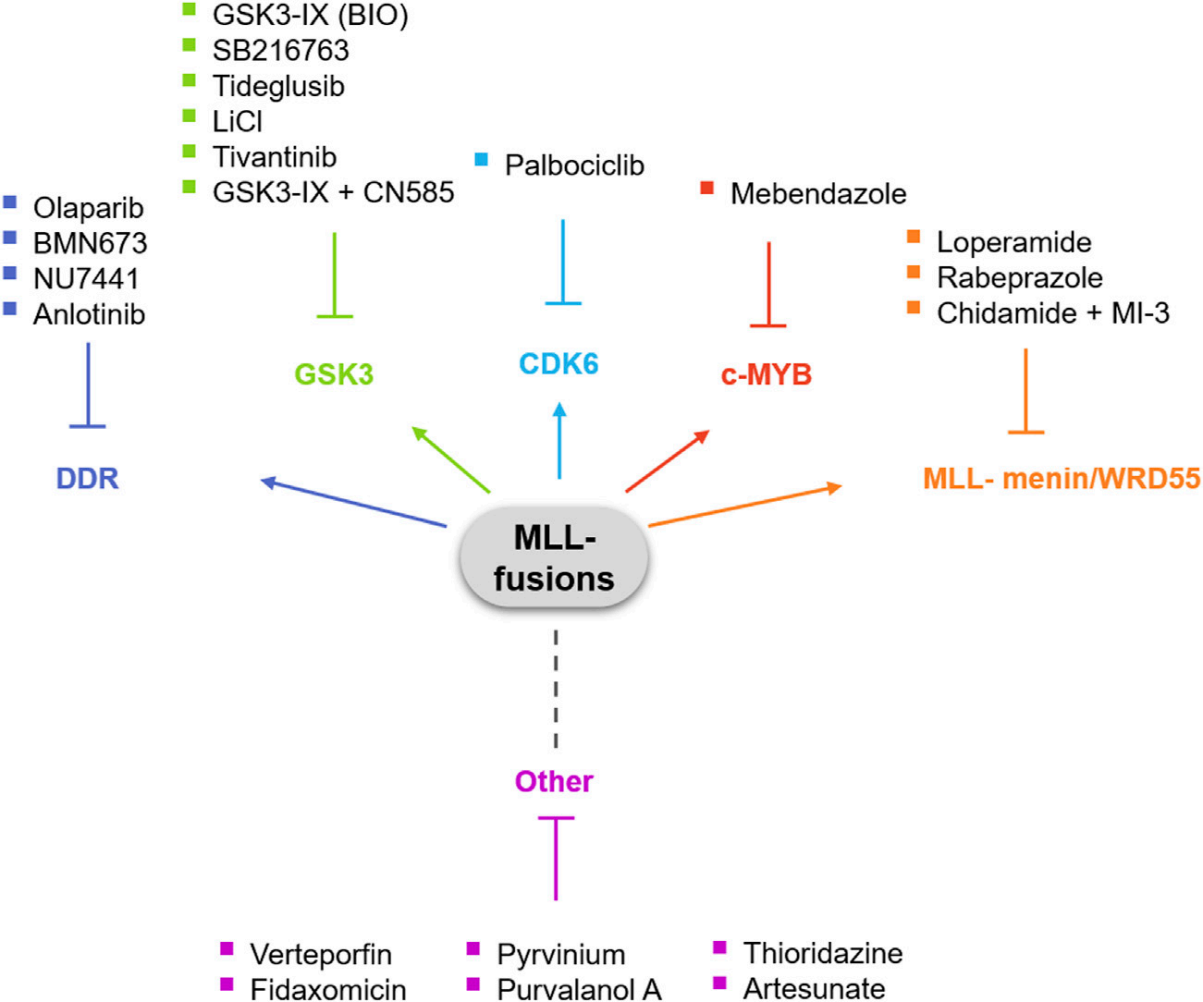
Opportunities for drug repositioning in MLL(KMT2A)-rearranged leukemia.

The significant rate of failure of the treatments currently used against acute leukemias with *MLL-*rearrangements is very frustrating and emphasizes the need for novel therapies that specifically target MLL fusion proteins and/or the downstream pathways these fusions dysregulate. In this review, we aim to highlight the therapeutic opportunities of drug repurposing for acute leukemias with *MLL-*rearrangements (Figure 1).

## Hypothesis Driven and Target-Based Strategies for Drug Repurposing in *MLL*-Rearranged Leukemia

The understanding of the molecular mechanisms and main targets of MLL fusion proteins can lead us to form hypotheses for the direction of therapeutic intervention. Besides developing *de novo* drugs acting on the pathways that are known to be dysregulated or implicated in the phenotype of leukemia cells with *MLL-*rearrangements, we can also get inspired and redirect drugs already targeting those pathways in other conditions towards leukemia treatment. Selective inhibitors can be repositioned to different biological systems based on their activity on their target or even because of their off-target effects. This provides an opportunity to take advantage of the knowledge we already have of MLL-fusion function and quickly test compounds and drugs available in the lab or the clinic.

### GSK3 Inhibitors

Glycogen synthase kinase 3 (GSK3) is a serine/threonine kinase with multiple roles in several signaling pathways in physiological and pathological conditions. GSK3 is involved in mRNA transcription, cell cycle, apoptosis, cell fate determination and stem cell maintenance (Beurel et al., 2015). As several of these pathways are implicated in disease pathogenesis, it comes as no surprise that GSK3 specific inhibitors have been in development for years. In hematopoiesis, GSK3 is important for the activity of hematopoietic stem cells and mice treated with GSK3 inhibitors show enhanced hematopoietic stem cell repopulation following bone marrow transplantation (Trowbridge et al., 2006). Importantly, inhibition of GSK3 showed potential as a treatment for *MLL*-rearranged leukemia, as in a small-scale screen aiming to identify compounds that could induce the growth arrest of genetically defined subsets of leukemia cells, AML and ALL cell lines that expressed MLL-AF4 or MLL-AF5 showed enhanced sensitivity to GSK3-IX (also known as BIO), a GSK3 inhibitor that also targets cyclin-dependent kinases (CDKs) and has been extensively used for the maintenance of pluripotency in human and murine embryonic stem cells (ESCs) (Tseng et al., 2006; Wang et al., 2008). Cell cycle analysis of AML and ALL *MLL*-rearranged cells treated with SB216763, another GSK3 inhibitor with therapeutic properties for pulmonary inflammation and fibrosis (Gurrieri et al., 2010), revealed reduction of G1-S phase progression. The response of *MLL*-rearranged cells to GSK3 inhibitors is mediated by p27^Kip1^, since the knockdown of the protein rescued the effects of GSK3 inhibition on cell cycle progression (Wang et al., 2008). The specificity of GSK3 inhibition in cells with *MLL-*rearrangements but not wild type cells is supported by studies that have shown that in *MLL*-rearranged cells, GSK3 regulates the expression of *Homeobox* (*HOX*) genes *via* CREB phosphorylation, leading to the induction of MEIS1-related gene expression and the subsequent maintenance of a stem cell-like phenotype in *MLL*-rearranged cells (Wang et al., 2010; Yeung et al., 2010). In addition, although *MLL*-rearranged leukemic stem cells (LSCs) were found to be resistant to GSK3 inhibition by lithium chloride (LiCl), an inhibitor that is approved by the Food and Drug Administration (FDA) for the treatment of epilepsy and bipolar disorder (Abu-Baker et al., 2013), they could be sensitized by β-catenin knockdown, indicating possible avenues for novel combination therapies (Yeung et al., 2010).

### DNA Damage Response Inhibitors

In *MLL*-rearranged leukemia, the DNA damage response (DDR) machinery is perturbed. Some MLL-fusion proteins are suggested to predispose leukemia cells to DNA damage and ultimately cause short latency (Eguchi et al., 2006). Also, although p53, a protein usually activated to protect the cells in response to DNA damage by inducing cell cycle arrest and apoptosis (Aylon and Oren, 2007) is not often mutated in *MLL*-rearranged leukemia, MLL fusion proteins have been shown to suppress p53 and the p53-mediated response to DNA damage (Wiederschain et al., 2005). Importantly, the DDR machinery is activated during MLL-ENL-oncogene-induced leukemic transformation and is important for the maintenance of stem cell-like properties (Takacova et al., 2012). Finally, in B-ALL with *MLL*-rearrangements, DDR is required for cells with oncogene-driven replicative stress (Chu et al., 2017). Therefore, it appears that the pharmacologic targeting of the DDR pathway has the potential of inducing specific synthetic lethality in leukemic cells with *MLL-*rearrangements.

A group of drugs currently redirected for leukemia therapy are the poly (ADP-ribose) polymerase (PARP) inhibitors. Poly (ADP-ribosyl)ation of nuclear proteins supports the survival of cells with low DNA repair capacity. The role of PARP proteins has been well established in *BRCA1/2* mutant tumors, that lack the mechanisms for homologous recombination. However, more studies have revealed new roles of PARP for gene transcription, protein stabilization and modulation of chromatin formation (Kraus and Hottiger, 2013; Krietsch et al., 2013). PARP inhibitors regulate critical functions of PARP proteins mainly in DNA-repair mechanisms such as single-strand breaks (SSBs) in base excision repair (BER) and homologous recombination-mediated double-strand break (DSB) repair (Haince et al., 2007; Haince et al., 2008; Bryant et al., 2009). Olaparib, rucaparib, niraparib and talazoparib are four PARP inhibitors that have been approved by the FDA for the treatment of germline *BRCA*-mutated breast or ovarian cancers (Plummer et al., 2005; Evers et al., 2008; Sandhu et al., 2010; Shen et al., 2013). The repurposing of PARP inhibitors to other malignancies has not been as successful as their use in breast and ovarian cancers. In hematological malignancies, inhibiting the activity of PARP proteins is very relevant, as these proteins regulate components often dysregulated in blood cancers, such as *ATM*, *ATR*, *CHK1*, and *RAD51* (Zhao and So, 2016). Importantly, MLL-AF9 expressing leukemia cells depend on mechanisms that prevent the accumulation of DSBs in order to maintain proliferation (Santos et al., 2014; Maifrede et al., 2017). One study has shown that *Parp1* ^*−/−*^ mouse bone marrow cells (mBMCs) expressing MLL-AF9 show reduced colony formation activity in comparison with *Parp1* ^*+/+*^ counterparts. These cells were shown to be modestly sensitive to the PARP1 inhibitor olaparib. In the same study, syngeneic mice transplanted with murine leukemia cells expressing MLL-AF9 were either left untreated or were treated with doxorubicin plus cytarabine, the PARP inhibitor BMN673, or the combination of the two. The results demonstrate a synergistic effect of the PARP1 inhibitor with the cytotoxic drug combination, overcoming the resistance to olaparib (Maifrede et al., 2017). While in leukemia cells with AML1-ETO and PML-RARa fusion proteins inhibition of PARP leads to an accumulation of DNA damage that results in cell differentiation and cell death, leukemia cells expressing MLL fusion proteins exhibit resistance to inhibition of PARP (Yeung et al., 2010). In AML, the expression of MLL fusion proteins results in the expression of the homeodomain transcription factor HOXA9 among others (Krivtsov and Armstrong, 2007; Yip and So, 2013). HOXA9 serves as a prognostic factor associated with poor AML treatment response (Golub et al., 1999) and its suppression has been linked to drug resistance in glioblastoma (Costa et al., 2010). The hypothesis that MLL-AF9-transformed cells show resistance to PARP inhibition possibly *via* the activation of HOXA9 was tested by Esposito et al. Inhibition of PARP reduced the colony formation ability of MLL-AF9-tranformed cells, but not HOXA9-independent E2A-PBX–transformed control cells while inducing differentiation and senescence (Esposito et al., 2015). Importantly, *Hoxa9*-deficient MLL-AF9 cells were highly sensitive to olaparib treatment, highlighting the role of HOXA9 in leukemic growth and the resistance of the *MLL-*rearranged AML cells to PARP inhibition (Esposito et al., 2015). Taking into consideration that HOXA9 is not essential for normal development (So et al., 2004; Lawrence et al., 2005; Smith et al., 2011), PARP inhibitors would appear to have a great therapeutic potential in leukemia with *MLL-*rearrangements.

ATR and ATM activate the DDR pathway and recognize DNA single or double strand breaks (Hurley and Bunz, 2007). It has been shown that they support cancer cells that are under replicative stress, therefore their inhibition is promising (Weber and Ryan, 2015). Inhibition of ATR in *MLL-*rearranged leukemia has therapeutic potential, since in mice where ATR expression is reduced, MLL-ENL AML cells growth is affected. Schoppy et al. generated a genetic system where ATR expression was conditionally reduced to 10% of normal levels in adult mice and studied the effects of this reduction *in vivo* comparing normal tissues and cancer. Although the suppression of ATR did not affect normal bone marrow, the authors reported inhibition of MLL-ENL-driven AML among others (Schoppy et al., 2012). Interestingly, in contrast to its function in overt leukemia the DDR functions as a barrier in the early stages of leukemogenesis. Thus, in a study examining the potential of the inactivation of the DDR barrier in leukemogenesis, Takacova et al. showed that ATM and ATR inhibitors inactivate the DDR barrier and support leukemia progression in a tamoxifen-inducible MLL fusion mouse model (Takacova et al., 2012). At the same time, the genetic ablation of critical regulators of DNA damage response such as *MLL4*, *ATM* or *BRCA1* in *MLL-*rearranged cells induces leukemic differentiation (Santos et al., 2014). Importantly, it has been demonstrated that ATR and ATM inhibitors synergize with PARP inhibitors in response to DNA damage (Aguilar-Quesada et al., 2007; Yazinski et al., 2017). Possibly this combination could be successful against *MLL-*rearranged leukemia as well.

The DNA-dependent protein kinase (DNA-PK) is a serine/threonine protein kinase consisting of a catalytic subunit (DNA-PKcs) and a Ku heterodimer that is made up of the Ku70 and Ku80 subunits and it belongs to the phosphatidylinositol 3-kinase (PI3K)-related kinase protein family (Hartley et al., 1995). DNA-PK is required for repairing DSBs through non-homologous end-joining (NHEJ) (Rassool, 2003; Shrivastav et al., 2008). In addition to its role in NHEJ, DNA-PK also cooperates with ATR and ATM to regulate DNA damage checkpoints and thus, cells that are ATM-deficient, heavily depend on DNA-PK (Meek et al., 2008; Riabinska et al., 2013; Tsuji et al., 2017). In cancers where ATM is dysregulated, inhibition of DNA-PK has a therapeutic potential. For example, CC-115, a selective dual inhibitor of the mammalian target of rapamycin (mTOR) kinase and DNA-PK, has been shown to effectively inhibit proliferation and induce apoptosis in lymphoma, leukemia, breast cancer, hepatocellular carcinoma, head and neck cancer and lung cancer cell lines (Tsuji et al., 2017). In chronic lymphocytic leukemia (CLL) cells, the same inhibitor caused the inhibition of the DNA damage repair pathway and induced apoptosis and is currently under clinical trial for CLL (Thijssen et al., 2016; Tsuji et al., 2017). NU7441 is another DNA-PKi with interesting potential. NU7441 sensitizes cancer cells in B-cell CLL, breast cancer, non-small cell lung carcinoma and nasopharyngeal carcinoma (NPC) cell lines (Elliott et al., 2011; Ciszewski et al., 2014; Yanai et al., 2017; Dong et al., 2018). As we discussed earlier, leukemia cells with *MLL*-rearrangements also respond to ATM inhibitors. Therefore, the potential synergy between DNA-PK inhibitors and inhibitors of the DDR pathway such as ATMi and PARPi could be interesting.

SET domain-containing protein 1A (SETD1A) is a histone 3 lysine 4 (H3K4) methyltransferase and is one of the six MLL family members found in mammals (*MLL1*, *MLL2*, *MLL3*, *MLL4*, *SETD1A*, and *SETD1B*) (Chan and Chen, 2019). H3K4 methylation is a post-translational modification critical for the regulation and maintenance of gene expression during development and frequently mutated in cancer (Ernst et al., 2004; Kandoth et al., 2013; Bledau et al., 2014; Denissov et al., 2014). In breast cancer, SETD1A controls mitosis by regulating the H3K4 methylation of the promoters of mitotic genes. When *SETD1A* expression is knocked-down, the cells present severe mitotic defects and senescence (Tajima et al., 2019). In leukemia with *MLL*-rearrangements, SETD1A is essential for cell proliferation. The non-enzymatic domain of SETD1A interacts with cyclin K to control DNA damage response-related gene expression during the S phase of the cell cycle (Hoshii et al., 2018). In a recent study, Chen J. et al. used anlotinib, an inhibitor originally designed to target receptor tyrosine kinase (RTK) related to tumor vasculogenesis (Sun et al., 2016; Chen et al., 2021). Anlotinib has shown anti-tumor activity in non-small cell lung cancer and hepatocellular carcinoma (Han et al., 2018; He et al., 2018; Shen et al., 2018; Syed, 2018). Anlotinib treatment of *MLL*-rearranged leukemic cell lines suppressed growth and caused G2/M cell cycle arrest and apoptosis. An RNA sequencing study of cells exposed to anlotinib revealed that anlotinib treatment causes downregulation of genes critical for DDR, such as the genes encoding for the DNA polymerase delts proteins (POLDs) that act as response regulators of the DDR, that strongly correlated with SETD1A and Akt expression, suggesting an anti-leukemic activity of anlotinib by targeting SETD1A- and Akt-mediated DNA damage response in *MLL*-rearranged leukemia cells (Chen et al., 2021).

These studies highlight the potential of inhibiting molecular players of the DDR pathway in acute leukemias with *MLL-*rearrangements.

### Cyclin-Dependent Kinase (CDKs) Inhibitors

The cyclin-dependent kinase 6 (CDK6) is a critical regulator of cell-cycle progression. CDK6 and CDK4 are critical for normal hematopoiesis, as the simultaneous deletion of the two kinases leads to embryonic lethality in mice (Malumbres et al., 2004). In normal hematopoiesis, CDK6 plays a crucial role in regulating the exit of long-term hematopoietic stem cells (HSCs) from quiescence and contributes to maintenance of the HSC pool (Laurenti et al., 2015). Besides its role in cell cycle regulation, CDK6 also regulates transcription by interacting with transcription factors of the STAT and AP-1 family (Kollmann et al., 2013). In ALL and MDS, CDK6 is needed to antagonize p53 responses during transformation (Bellutti et al., 2018). One of the consequences of *MLL*-rearrangements is the expression of stem cell gene programs that support the leukemia-initiating activity of hematopoietic stem cells (Krivtsov et al., 2006). The importance of CDK6 for leukemia with *MLL*-rearrangements gained attention when Placke et al. identified CDK6 as selectively implicated in the growth of MLL-AF9, MLL-AF4, and MLL-AF6 rearranged cells compared to wild type controls by using a functional genetic RNA interference screening approach (Placke et al., 2014). It has been shown that in infant MLL-AF4 ALL and MLL-AF9 AML, *CDK6* but not *CDK4* is a target of the MLL fusion proteins (Placke et al., 2014). MLL-AF9 can bind the *CDK6* locus in wild type cells and subsequently increase the levels of CDK6. CDK6 then inhibits myeloid differentiation of AML cells while cell cycle progression is not affected (Placke et al., 2014; Van der Linden et al., 2014). The effect of CDK6 inhibition on myeloid differentiation might also be linked with the fact that CDK6 is a target of miRNA29a, a regulator of myeloid differentiation in hematopoietic progenitors and AML cells (Wang et al., 2013). The catalytic activity of CDK6 appears to be crucial for this effect, as treatment of the cells with the CDK6 inhibitor palbociclib rescues the effect of CDK6 overexpression by inducing myeloid differentiation (Placke et al., 2014). Palbociclib is an FDA-approved CDK4/6 selective inhibitor originally developed for the treatment of hormone receptor (HR)-positive and human epidermal growth factor receptor (HER2)-negative breast cancer patients (Rocca et al., 2014). The study by Placke et al. shows its great potential for reproposing against *MLL-*rearranged leukemias.

### MLL-Menin/WDR5 Interaction Inhibitors

The MLL-menin interaction is considered a requirement for the leukemogenic activity of MLL fusion proteins (Yokoyama et al., 2005; Caslini et al., 2007). Therefore, the *de novo* drug development of agents able to disrupt this interaction could be a successful strategy in *MLL*-rearranged leukemia. Li et al. showed that when the MLL-menin interaction is disrupted in mice, only the expression of a small number of genes is affected and importantly, these genes are not the critical downstream effectors of MLL for normal hematopoiesis or B lymphopoiesis. This indicates that menin is not required for the gene expression program regulating development of HSCs and B cells (Li et al., 2013). Therefore, the fact that menin is not required for MLL function during normal hematopoiesis makes drugs that target this interaction safe and selective for *MLL-*rearranged leukemia (Li et al., 2013; He et al., 2016). In this context, there have been successful *de novo* designed small molecule inhibitors of the MLL-menin interaction, such as members of the thienopyrimidine class (Shi et al., 2012; Senter et al., 2015; Borkin et al., 2016). The crystal structures of the inhibitors MI-2-2 and MIV-6R were used in scaffold hopping calculations that as a result suggested loperamide, an antidiarrheal drug used in acute and chronic diarrhea as well as in ileostomy, as a possible inhibitor of the interaction between MLL and menin (Yue et al., 2016). Similarly to molecules that disrupt the MLL-menin interaction, histone deacetylase (HDAC) inhibitors also affect the transcription-regulatory machinery governing the epigenetic changes that drive leukemogenesis. It seems, therefore, that there is a potential for synergy between MLL-menin interaction inhibitors and HDAC inhibitors. In a study using *MLL-*rearranged AML cells, the HDAC inhibitor chidamide, a member of the benzamide class that has been approved by the Chinese FDA for treatment of relapsed or refractory peripheral T cell lymphoma (PTCL), showed synergistic effects with the MLL-menin interaction inhibitor MI-3 on cell growth and apoptosis. The combination of the two agents was also effective in *in vivo* experiments with a mouse xenograft model established by subcutaneous inoculation with *MLL-*rearranged MOLM-13 cells (Ye et al., 2019). This study shows the opportunity of combining drugs developed *de novo* to target MLL with repurposed agents to achieve synergy and optimal results.

MLL-fusion proteins lack the C-terminal SET domain of wild-type MLL. Therefore, wild-type MLL is required for the fusions in order to regulate promoter H3K4 methylation and expression of target genes, such as *HOXA*/*MEIS1* (Milne et al., 2005; Thiel et al., 2010; Chen et al., 2017). Thus, the targeting of the H3K4 methyltransferase activity of MLL can be used to block the function of the MLL-fusion proteins in leukemia with MLL-rearrangements. This can be achieved by blocking the MLL and WDR5 protein-protein interactions (Grembecka et al., 2012; Cao et al., 2014). To this end, Chen et al. performed cell-based screenings with a compound collection to identify new therapeutic agents against *MLL-*rearranged leukemia cells and identified rabeprazole, a proton pump inhibitor used for heartburn, acid reflux and gastro-esophageal reflux disease (GORD), as a candidate for inhibiting the growth of *MLL-*rearranged cell lines but not *MLL* wild type cells (Chen et al., 2020). The treatment with rabeprazole caused the downregulation of the MLL fusion protein targets *HOAX9* and *MEIS1* and the H3K4me1 and H3K4me2 methylation levels, suggesting a dysregulation of the MLL histone methyltransferase activity. WDR5 is a key structural component of the histone methyltransferase activity of MLL (Dou et al., 2006; Karatas et al., 2013), therefore the authors tested the hypothesis that rabeprazole may exhibit its MLL-specific anti-leukemic effect *via* disruption of the protein-protein interaction between MLL and WDR5. By tagging the MLL win motif peptide, responsible for binding WDR5, with 5-FAM tracer as probe, they confirmed the inhibition of MLL-WDR5 interaction upon treatment with rabeprazole (Chen et al., 2020). Furthermore, they evaluated the potential of four more proton pump inhibitors for their anti-proliferation activity on leukemia cells and confirmed that proton pump inhibitors selectively inhibit leukemia cells with *MLL*-rearrangements and not wild type leukemia cells or normal control cells (Chen et al., 2020).

Given the complexity of the MLL recombinome and the large number of different MLL-fusion proteins (Meyer et al., 2018), only drugs targeting regions, pathways or mechanisms that are common to all, e. g., dugs targeting the N-terminus of MLL, will be effective across *MLL*-rearranged leukemia as a whole. It is also becoming clear that rare cases of non-canonical *MLL* fusions also occur; for example the recently described fusion incorporating PHD domains (Meyer et al., 2019). This also poses a challenge to therapies targeting conventional MLL-fusion proteins. The success and broad applicability of the later will depend on the effectiveness of them targeting common core pathways and interactions in *MLL*-rearranged leukemia. Predicting this will depend on further molecular dissection of these different fusions in the laboratory.

## Using *in Silico* Tools and Drug Screens to Identify Novel Drugs With Anti-Leukemic Therapeutic Potential

The gene expression and regulatory profiles that characterize a disease can be used for signature-based strategies for the identification of drugs that can repurposed. Tools such as the Connectivity MAP (CMAP), GWAS and the Library of Integrated Network-based Cellular Signatures (LINCS) can be used among others for signature-based screening (Welter et al., 2014; Koleti et al., 2018). These tools open opportunities for the discovery of new drug-target combinations and significantly expand the potential use of already available therapeutic agents minimizing the time and cost of the research and development phase.

### Mebendazole

The transcription factor c-MYB plays a central role in definitive hematopoiesis and the development of multiple hematopoietic lineages (Pattabiraman and Gonda, 2013). In leukemia, c-MYB-regulated transcriptional programs have been demonstrated as essential for the initiation of the disease as well as the self-renewal and maintenance of leukemia cells with *MLL*-rearrangements (Hess et al., 2006; Somervaille et al., 2009; Zuber et al., 2011). Importantly, reduced c-MYB levels are still compatible with normal hematopoiesis, therefore inhibition of c-MYB appears to have a therapeutic opportunity for *MLL-*rearranged leukemia (Emambokus et al., 2003; Zuber et al., 2011). However, its nature as a transcription factor makes it difficult to target with small molecule inhibitors and emphasizes the need for alternative targeting approaches. For example, celastrol, a natural compound that targets the interaction of c-MYB with the transcriptional co-activators CBP/P300 has shown promising anti-leukemia activity in AML (Uttarkar et al., 2016). Similarly, peptidomimetics that achieved the inhibition of the assembly of the c-MYB-CBP/P300 complex also caused leukemia cell growth arrest and extended survival of immunodeficient mice engrafted with *MLL*-rearranged leukemia cells (Ramaswamy et al., 2018). Surprisingly, the treatment of AML cells with mebendazole, an anti-helminth drug routinely used in the clinic for children with parasitic infections, has shown anti-leukemia activity *via* c-MYB inhibition in both *MLL*-rearranged and non-rearranged cells (Walf-Vorderwülbecke et al., 2018). Mebendazole was identified as the top hit of in a study that used the CMAP database, that offers a large reference catalogue with the gene expression profiles of various human cells stimulated with different chemicals. The CMAP database was screened with a c-MYB gene expression signature derived from integrating MLL fusion protein specific gene expression changes with a list of previously published direct c-MYB target genes (Walf-Vorderwülbecke et al., 2018). This illustrates the possibility of *MLL*-rearranged directed drug repurposing approaches leading to the identification of therapeutic approaches that have broad AML applicability. This is supported by recent studies confirming the importance of c-MYB in a broad range of AML subtypes (Armenteros-Monterroso et al., 2019; Takao et al., 2021) (REFs–Takao et al. DOI 10.7554/eLife.65905; Armenteros-Monterroso et al. DOI: 10.1038/s41375-019-0495-8).

### Verteporfin

The CMAP database was used in another study for the identification of genes that are exclusively up- or downregulated in LSCs, the self-renewing cell population that is required for the initiation and maintenance of AML (Dick, 2005; Xiu et al., 2018). The screening identified verteporfin as an agent with potential anti-AML activity. Verteporfin has been approved by the FDA in photodynamic therapy to eliminate abnormal blood vessels in macular degeneration. It has also been used in ALL, where its anti-leukemic activity has been studied in respect to its minimal effects on normal hematopoiesis (Gibault et al., 2016; Morishita et al., 2016). Xiu et al., treated AML cells with verteporfin and found that it suppressed cell growth *in vitro* and delayed the development of the disease *in vivo* (Xiu et al., 2018). They attributed the effects of verteporfin to the upregulation of the expression of the components of the non-canonical NFκB signaling. This is particularly important for leukemia with *MLL-*rearrangements, where RUNX1 can interact with MLL and NFκB. This interaction allows the non-canonical signaling component to have an anti-MLL activity by interfering with the RUNX1-MLL complex (Koh et al., 2013).

### Fidaxomicin

In one study aiming to identify vulnerabilities in HSC-derived human *MLL*-rearranged AML cells resistant to chemotherapy, Zeisig et al. identified genes differentially expressed in *MLL*-rearranged HSC and common myeloid progenitor (CMP) cells and compared them with the Genomic Element Associated with drug Resistance (GEAR) database (Wang et al., 2017). The screening came up with two genes associated with resistance to doxorubicin treatment; *IL6*, encoding for interleukin-6 and *ABCC3*, a member of the ABC family of organic anion transporters (Zeisig et al., 2021). Intriguingly, ABCC3 overexpression has been associated with chemoresistance and poor prognosis in both pediatric and adult AML (Steinbach et al., 2003; Benderra et al., 2005) and Zeisig et al. confirmed its higher expression in *MLL*-rearranged HSC cells and thus revealed the translational potential of targeting ABCC3 in *MLL-*rearranged leukemia (Zeisig et al., 2021). ABCC3 is targeted by fidaxomicin, a drug used for diarrhea associated with *Clostridium difficile* infection (Golan and Epstein, 2012). Fidaxomicin treatment of *MLL*-rearranged HSC cells inhibited cell growth *in vitro* and reduced tumor burden when combined with doxorubicin in an NSG xenograft model transplanted with HSC-derived MLL-AF6 cells (Zeisig et al., 2021). The fact that the combination treatment had significantly higher effects in the reduction of the tumor burden in the animal models that the authors used suggests that the repurposing of fidaxomicin to target ABCC3 in leukemia acts synergistically with the *de novo* drugs to overcome chemoresistance in *MLL-*rearranged leukemia.

### Pyrvinium

With a different approach using a high-throughput drug library screening methodology, more than 4,000 compounds were screened at various final concentrations on two primary AML patient samples with MLL-AF9 translocation aiming to identify drugs that can achieve inhibition of more than 50% leukemic cell viability. Pyrvinium was the highest-ranked hit at the highest dose tested and achieved elimination of the leukemic cells in both samples, while affecting the viability of non-leukemic bone marrow control samples by 50% (Wander et al., 2021). Pyrvinium is an FDA approved anthelmintic drug used in the clinic for the treatment of pinworms in humans (Beck et al., 1959). Anti-cancer effects of pyrvinium have been reported in the past, with two mechanisms of action usually suggested; inhibition of the canonical Wnt signaling pathway *via β*-catenin degradation (Xu et al., 2018) and mitochondrial respiration impairment (Xiang et al., 2015; Xiao et al., 2016). Although the authors did not investigate in detail the mechanism of the anti-leukemia activity of pyrvinium in the MLL samples, they showed lack of *β*-catenin protein in *MLL-*rearranged AML cells and, at the same time, mitochondrial localization of the drug in the MLL cells. Therefore, they speculate that pyrvinium might cause mitochondrial respiration impairment in leukemia cells with *MLL* translocations (Wander et al., 2021).

### Thioridazine and Artesunate

In a high-throughput drug screening of 1,280 compounds, 104 were identified as active against MLL-AF6 rearranged cell lines, with 20 of them showing activity specifically in *MLL-*rearranged cells and 10 exclusively on the t (6;11)-rearranged cells (Tregnago et al., 2020). One of those hits was thioridazine, an anti-psychotic agent primarily used for schizophrenia. Thioridazine caused apoptosis and cell cycle arrest exclusively in the t (6;11) AML cells. Importantly, thioridazine sensitized non- t (6;11)-rearranged cells when they were transfected with an MLL-AF6 expressing vector. The anti-leukemia effects of thioridazine were also tested *in vivo*, where the treatment significantly reduced the progression of tumor growth in t (6;11) SHI-1 cell transplanted mice, but not in mice transplanted with t (5;17) HL60 or t (9;11) THP-1 cells, highlighting the specificity of thioridazine against cells with the *MLL-AF6* fusion. To understand the mechanism behind the MLL-AF6 specific anti-leukemia activity of thioridazine, the authors used a large-scale method combining quantitative proteomics with affinity enrichment and identified the calcium binding proteins S100A8 and S100A9, and ANXA6, a component of the annexin family, as targets of thioridazine. These three proteins are known to form a complex associated with cytoskeletal filaments (Bode et al., 2008). Therefore, the authors investigated the hypothesis that thioridazine treatment may induce cytoskeletal changes in the MLL-AF6 expressing cells and confirmed that morphologic changes and accumulation of large F-actin aggregates were induced upon exposure to thioridazine (Tregnago et al., 2020). The treatment with thioridazine caused an influx of external Ca^2+^ possibly *via* the cytoskeletal rearrangement caused by the drug. This Ca^2+^ overload led to reactive oxygen species (ROS) overproduction that was detrimental for the t (6;11) AML cells (Tregnago et al., 2020). Intriguingly, another class of drugs that leads to cellular and mitochondrial ROS accumulation in *MLL*-rearranged cells are the artemisinins, a family of antimalarial compounds. In their study, Kumar B. et al., treated *MLL-*rearranged AML cells expressing MLL-AF9 with artesunate and confirmed its cytotoxic activity *in vitro* and *in vivo* as well as its synergy with the BCL-2 inhibitor venetoclax. Mechanistically, the authors linked the activity of artesunate with the loss of the mitochondrial membrane potential and the generation of ROS (Kumar et al., 2017).

### ABT-737 and Purvalanol A

In a study using an alternative multiplex screening approach, Lappin et al. screened a library of 80 apoptosis-inducing agents for potential combinations with compounds with therapeutic potential for pediatric AML (Lappin et al., 2020). The screening identified ABT-737, a B-cell lymphoma (BCL)-family inhibitor and Purvalanol A, a CDK2 inhibitor, as a combination with therapeutic potential for *MLL*-rearranged leukemia and internal tandem duplications of the fms-related tyrosine kinase 3 gene (FLT3-ITD). This high-throughput screening approach proved very effective, as it combined the selection of a therapeutically highly relevant drug library as step one, and then the application of an all-pairs testing algorithm for identification of novel partners for combination treatments. This methodology achieves the reduction of consumables and time in the laboratory and opens up opportunities identification of novel combination therapies including the repurposing of therapeutic agents (Lappin et al., 2020).

## Conclusion

The understanding of the network of gene expression regulation driven by MLL fusion proteins in acute leukemia has given us a better insight of the molecular mechanisms that these fusion proteins hijack in order to initiate and maintain leukemogenesis. The protein complexes that cooperate with MLL fusion proteins and the subsequent gene expression changes open up numerous opportunities for pharmacological targeting specific to the leukemia cells with *MLL-*rearrangements. The successful modelling of *MLL*-rearranged acute leukemia has also provided fruitful experimental strategies in the search for new therapies. In this direction, we can take advantage of the technologies used for drug repurposing and identify therapeutic agents with the potential to specifically target leukemia cells with *MLL*-rearrangements. Exploring drug repurposing provides a new toolkit to tackle the challenges of clinical translation. The opportunity of applying known therapeutic strategies against new targets by repurposing drugs in the clinic is developing into a core strategy in drug development.
